# Supplementary material for: Targeting Endometrial Cancer Stem Cell Activity with Metformin Is Inhibited by Patient-Derived Adipocyte-Secreted Factors
Source: Cancers (Basel). 2019 May 11;11(5):653. doi: 10.3390/cancers11050653 (PMC6562824; doi:10.3390/cancers11050653)
Supplement: Supplementary file 1 [file cancers-11-00653-s001.pdf]

Supplementary materials: Targeting Endometrial Cancer Stem Cell Activity with Metformin Is Inhibited by Patient-Derived Adipocyte-Secreted Factors

Sarah J. Kitson, Matthew Rosser, Deborah P. Fischer, Kay M. Marshall, Robert B. Clarke and Emma J. Crosbie

Supplementary Methods

Endometrial Cancer Cell Lines

Ishikawa cells represent a well differentiated cell line, expressing oestrogen and progesterone receptors, and have wild-type K-Ras and were purchased from HPA Culture Collection (Salisbury, UK) [1]. Hec-1a is a moderately differentiated cell line with defective oestrogen receptors, activating K-Ras mutations and positive PTEN expression and was obtained from ATCC (Middlesex, UK) [1].

Adipocyte Culture

The omental tissue was washed twice in phosphate buffered saline (PBS) to remove excess blood. Tissue was finely dissected into 1–2 mm^3 pieces and placed in a centrifuge tube containing collagenase/dispase dissolved in pre-adipocyte growth media to give a final concentration of 1mg/mL. The tissue suspension was placed on a flask shaker for two hours at 37 °C to aid digestion before being filtered through a 100 μm filter to remove connective tissue and undigested fat. Following centrifugation, the cell pellet was resuspended in pre-adipocyte growth media and transferred to a humidified incubator for culture.

Oil Red O staining was used to detect intracellular lipids as confirmation of attainment of a mature adipocyte phenotype. A working solution of three parts Oil Red O stock solution (Sigma-Aldrich, Dorset, UK) diluted with two parts distilled water was made and filtered through a 0.2 μm syringe filter to remove any debris. Growth media was aspirated from wells containing confluent mature adipocytes and 10% formaldehyde (w/v) in PBS was added and left for 10 minutes at room temperature for fixation to occur. Cells were subsequently washed twice with PBS before the working solution of Oil Red O was applied and left for 15 minutes. Residual extracellular stain was removed by repeated washing with distilled water before cells were visualised under a light microscope.

Sphere formation and passaging

A single cell suspension was created by manual disaggregation (25 gauge needle) and either 5000 (Ishikawa) or 2000 (Hec-1a) cells were seeded in each well of a poly-HEMA (poly (2-hydroxyethylmethacrylate)) coated six well plate in stem cell media (phenol-red free, high glucose DMEM/F12 [Gibco, Paisley, UK] with 1% (v/v) penicillin/streptomycin, 20 ng/mL epidermal growth factor (EGF) and 10ml B27 [Gibco, Paisley, UK]) in the presence or absence of metformin (Sigma-Aldrich, Dorset, UK). Cells were cultured for five days before spheres with a diameter >50 μm were counted at ×40 magnification using a light microscope. Sphere formation efficiency (SFE) was calculated by dividing the number of spheres formed by the number of single cells initially plated. To assess self-renewal, spheres were counted, mechanically disrupted, centrifuged (450 g) and dissociated through the addition of trypsin (Sigma-Aldrich, Dorset, UK) for 90 seconds. Single cells were re-plated at the original seeding density. The self-renewal capacity was determined by dividing the number of secondary spheres formed after five days in culture by the total number of primary spheres.

Sulforhodamine B (SRB) Cytotoxicity Assay

Cells were plated at a density of 1000 cells/well or 10,000 cells/well (in the case of conditioned media experiments where cell proliferation was markedly reduced due to the omission of FBS from the growth media). After 24 hours, growth media was either replaced with fresh growth or conditioned media in the presence or absence of metformin and maintained in culture for four (conditioned media experiments) or five days. Cells were then fixed with 10% (v/v) trichloracetic acid for one hour at 4–8°C before being washed and left to dry overnight. The following day, 0.4% SRB (Sigma-Aldrich, Dorset, UK) was added and left for 15 minutes at room temperature before unbound stain was removed by washing with 1% (v/v) acetic acid. Bound protein was solubilised with 10 mM Tris and absorption measured at 450 nm.

Real Time quantitative-Reverse Transcriptase-Polymerase Chain Reaction (RT-qPCR) Quantification and quality Assurance of Extracted RNA was performed using the Agilent 2100

Bioanalyzer and Qubit RNA HS Assay Kit (Fisher Scientific, Loughborough, UK).

Reverse transcription of extracted RNA was performed on a thermal cycler (PTC-200 Thermal Cycler, MJ Research, Minnesota, USA) for 5 minutes at 25 °C, 30 minutes at 42 °C and 5 minutes at 80 °C.

Conditions for pre-amplification were 10 minutes at 95 °C, 15 cycles of 15 seconds at 95 °C and 4 minutes at 60 °C. After cycling, the reaction was diluted 1:5 using DNA suspension buffer (Teknova, Hollister, USA) and stored at −20 °C until required.

Assay stocks (×10) for the RT-qPCR were prepared using the Taqman gene expression assays of interest and a house keeper gene, phosphoglycerate kinase 1 (PGK1), diluted in Assay Loading Reagent (PN 100-5359, Fluidigm, San Francisco, UK). RT-qPCR was performed using a thermal mix consisting of 30 minutes at 25 °C, 60 minutes at 70 °C and 2 minutes at 50 °C followed by a Hot Start of

1 minute at 95 °C and 35 cycles of 5 seconds at 96 °C and 20 seconds at 60 °C.

Fold change was calculated using the formula:

fold change=2-ΔΔCt

where Ct is the threshold cycle, ΔCt=Ct of the specific gene-Ct of the housekeeper gene PGK1 and ΔΔCt=ΔCt of $ALDH^{high}/CD133^{+ve}$ cells-ΔCt of $ALDH^{low}/CD133^{-ve}$ cells

Supplementary Tables and Figures

Table S1. Taqman assays used for gene expression analysis.

Gene symbol	Gene Name	Assay ID
SOX2	*SOX2*	Hs01053049_s1
NANOG	*NANOG*	Hs02387400_g1
BMI1	*BMI1*	Hs00995520_g1
HEY1	*HEY1*	Hs01114113_m1
HES1	*HES1*	Hs00172878_m1
WNT2	*WNT2*	Hs00608224_m1
CTNNB1	*B-CATENIN*	Hs00355045_m1
YAP1	*YAP1*	Hs00902712_g1
TWIST1	*TWIST*	Hs01675818_s1
SNAI1	*SNAI1*	Hs00195591_m1
ZEB1	*ZEB1*	Hs01566408_m1
CDH1	*E-CADHERIN*	Hs01023895_m1
OCLN	*OCCLUDIN*	Hs00170162_m1
CLDN3	*CLAUDIN*	Hs00265816_s1
DSP	*DESMOPLAKIN*	Hs00950591_m1
EPCAM	*EPCAM*	Hs00901885_m1
VIM	*VIMENTIN*	Hs00958111_m1
CDH2	*N-CADHERIN*	Hs00983056_m1
PGK1	*PGK1*	Hs00943178_g1

Table S2. Characteristics of patients from whom omental biopsies were obtained for primary pre-adipocyte culture and maturation.

Patient ID	Age	BMI	Presence of Diabetes	Grade of Endometrial Cancer	FIGO Stage of Endometrial Cancer	Trial Treatment Allocation
C21	59	54.2	No	1	1a	Metformin
O15	53	36.6	No	2	3a	Metformin
S40	51	25.3	No	3	2	Placebo
W06	64	29.4	No	1	1a	Metformin
S33	61	41.4	No	1	2	Placebo
C27	78	28.5	No	3	1b	Placebo

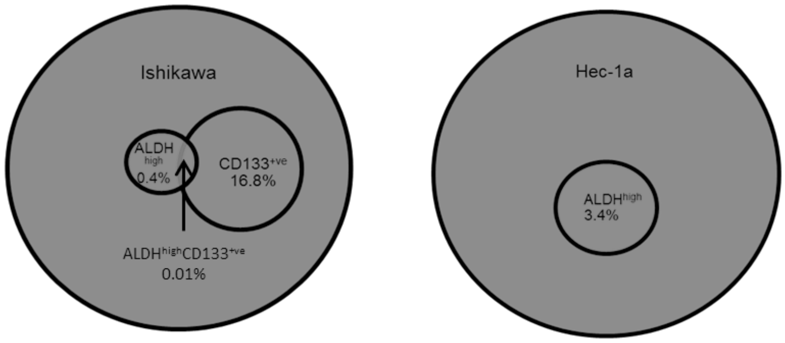

Figure S1. Relationship between ALDH^{high} and CD133^{+ve} cells in Ishikawa and Hec-1a cell lines (n = 5).

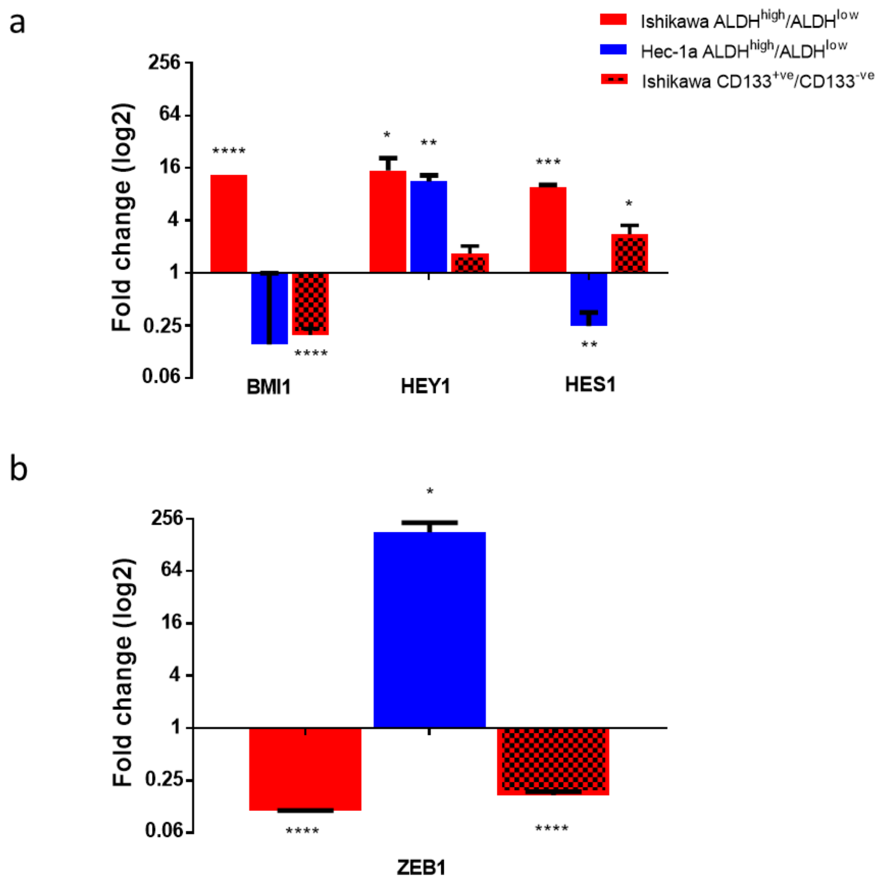

Figure S2. Expression of genes associated with a cancer stem cell phenotype in ALDH^{high} and CD133^{+ve} endometrial cancer cells. **(a)** RT-qPCR of genes associated with pluripotency and self-renewal in ALDH^{high} and CD133^{+ve} cells (n = 3). **(b)** RT-qPCR of EMT transcription factor genes in ALDH^{high} and CD133^{+ve} cells (n = 3).

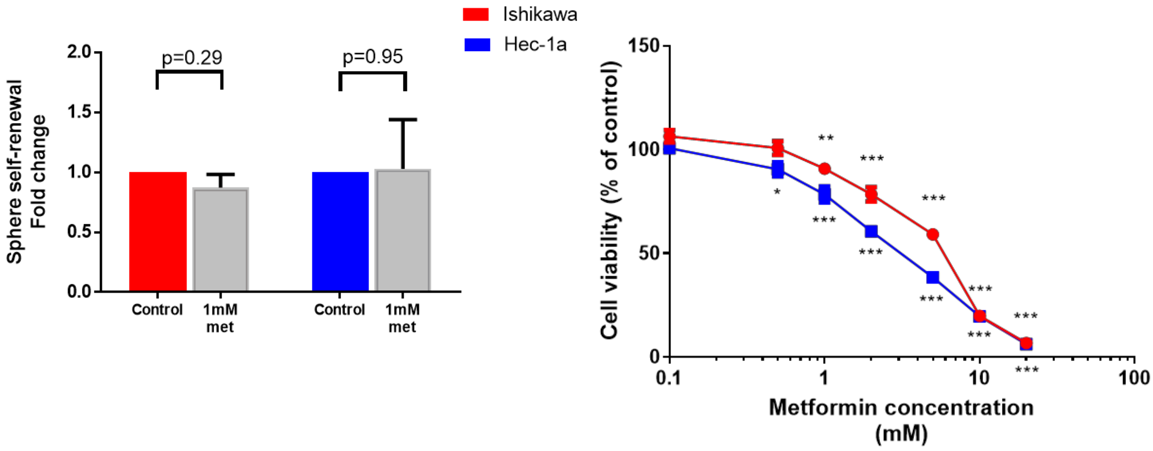

(a) (b)

Figure S3. Effect of metformin on endometrial cancer stem cell number and activity. **(a)** Self-renewal of sphere-initiating Ishikawa and Hec-1a cells treated with metformin in the first generation (n = 4). **(b)** Sulforhodamine B (SRB) cytotoxicity assay of Ishikawa and Hec-1a cells treated with metformin (n = 3).

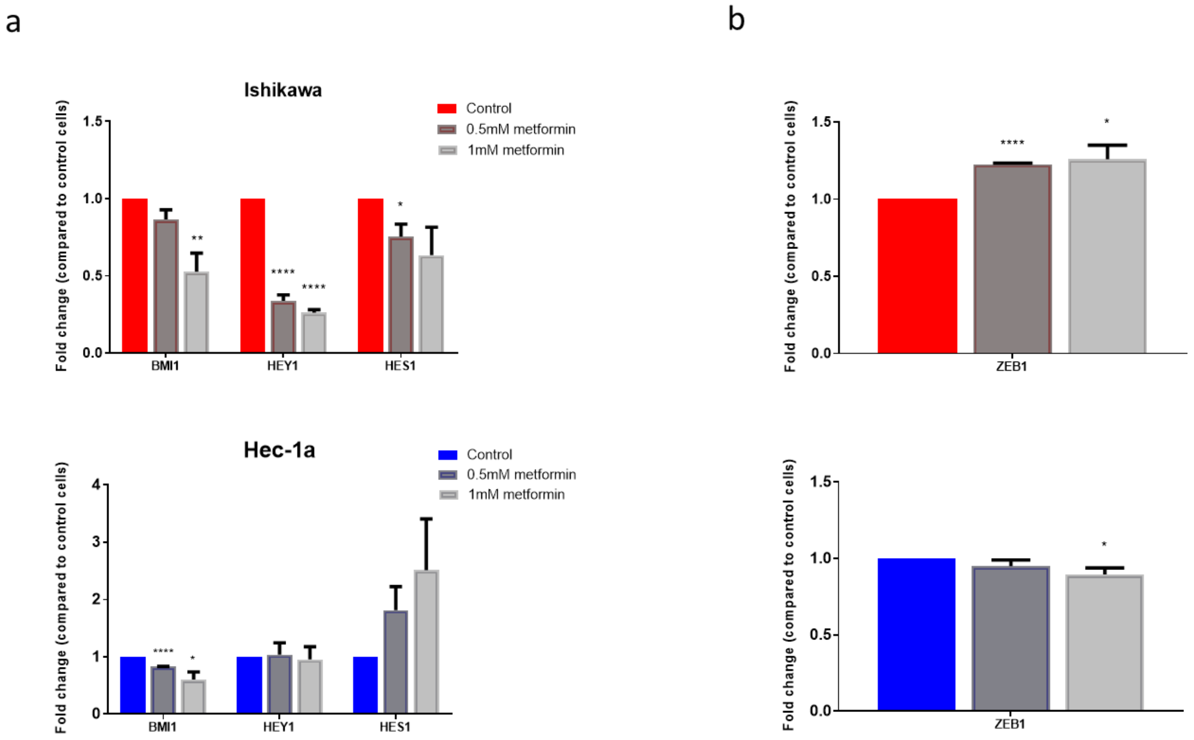

Figure S4. Effect of metformin on stem cell and EMT gene expression. **(a)** On the top, qRT-PCR of genes within the Wnt and Hippo signalling pathways in Ishikawa cells treated with metformin. Underneath, qRT-PCR of the same genes in Hec-1a cells treated with metformin (n = 3). **(b)** On the top, qRT-PCR of EMT transcription factor genes in Ishikawa cells treated with metformin. Underneath, qRT-PCR of. the same genes in Hec-1a cells treated with metformin (n = 3). Data are represented as the mean ±SEM. * $p \leq 0.05$, ** $p \leq 0.01$, *** $p \leq 0.001$, **** $p \leq 0.0001$.

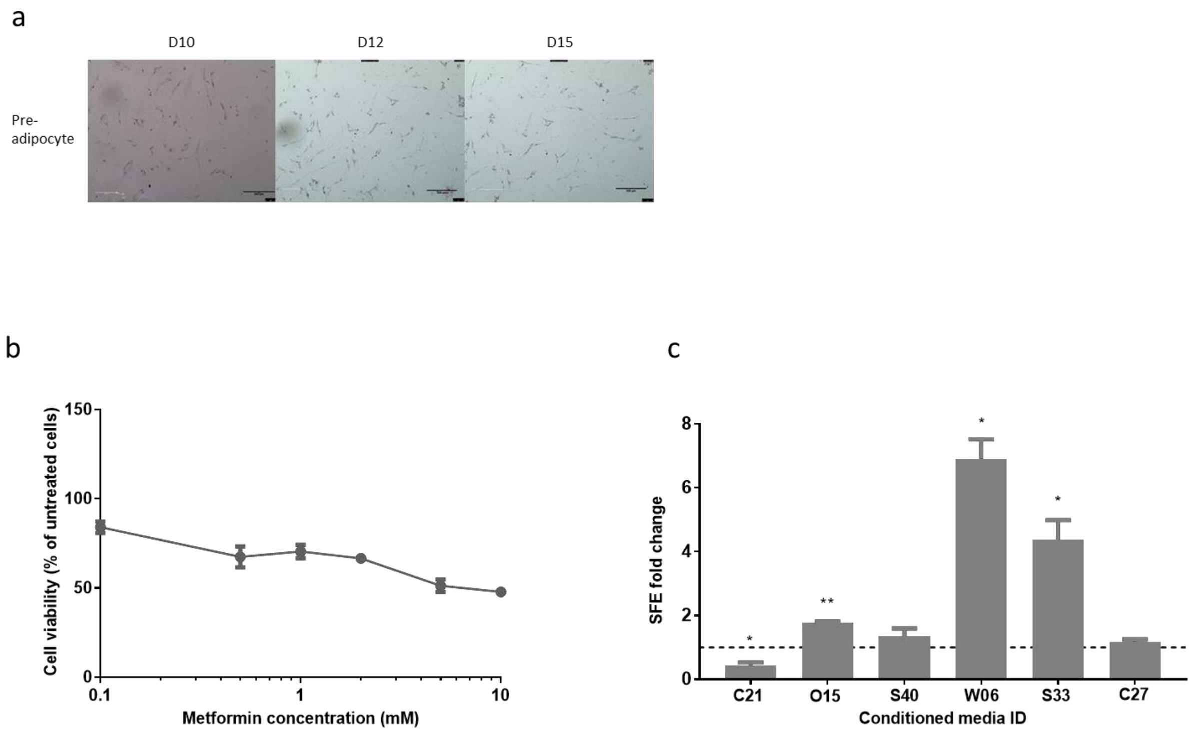

Figure S5. Effect of pre-adipocyte conditioned media (PACM) on endometrial cancer stem cell activity and response to metformin. **(a)** images at ×100 magnification of pre-adipocytes exposed to control growth media for 15 days before being stained with Oil Red O. **(b)** SRB cytotoxicity assay of Ishikawa cells exposed to PACM and treated with metformin (n = 4). **(c)** SFE of Ishikawa cells treated with PACM (n = 6).

References

1. Iglesias, D.A.; Yates, M.S.; van der Hoeven, D.; Rodkey, T.L.; Zhang, Q.; Co, N.N.; Burzawa, J.; Chigurupati, S.; Celestino, J.; Bowser, J.; et al., Another surprise from metformin: Novel mechanism of action via k-ras influences endometrial cancer response to therapy. *Mol. Cancer Ther.* **2013**, *12*, 2847–2856.

© 2019 by the authors. Licensee MDPI, Basel, Switzerland. This article is an open access article distributed under the terms and conditions of the Creative Commons Attribution (CC BY) license (http://creativecommons.org/licenses/by/4.0/).
